# Non-haematologic alloimmune manifestations following paediatric haematopoietic stem cell transplantation: a case series

**DOI:** 10.1007/s00277-026-07252-1

**Published:** 2026-09-07

**Authors:** Hilary Cahill, Ramya Hanasoge-Nataraj, Oana Mirci-Danicar, Omima Mustafa, Robert F. Wynn, Srividhya Senthil

**Affiliations:** Department of Paediatric Haematology and Bone Marrow Transplant, Manchester University NHS Foundation Trust, Manchester, UK

**Keywords:** Haematopoietic stem cell transplantation, Alloimmunity, Immune reconstitution, Alloimmune manifestations, Pediatric

## Abstract

Non-haematological alloimmune manifestations following haematopoeitic stem cell transplant are rare, but contribute to significant morbidity and mortality in paediatric transplant recipients. Current literature is limited to case reports of individual patients. Often, diagnosis can be challenging and conditions require multiple lines of therapy. We describe three unique cases of non-haematological alloimmune manifestations from a single centre in the UK, which demonstrates the breadth of alloimmunity in paediatric transplant patients from isolated single organ disease, to widespread systemic alloimmune disease necessitating second haematopoietic stem cell transplant. Early consideration of such manifestations is essential to facilitate appropriate diagnosis and initiation of targeted therapies to improve patient outcomes.

## Introduction

Alloimmune manifestations following haematopoetic stem cell transplant (HSCT) are increasingly recognised as a contributor to morbidity and mortality in HSCT recipients, and are likely under-recognised. Such manifestations are distinct from the more common immune-mediated acute and chronic graft-vs-host disease, and may involve a single organ or present in a multi-system manner. Often, clinical features may overlap with more common post-transplant complications and be difficult to identify and diagnose. The literature is sparse and limited in paediatric patients to case reports, with described patients experiencing single organ alloimmune involvement, typically haematologic, and often requiring many lines of therapy. The pathophysiology is poorly understood and thought to be due to the interaction between the dysregulation of a recovering immune system, and inflammatory factors. Non-haematological alloimmune manifestations are presumed to be driven by donor immunity against host organs and tissues [1].

We describe three interesting cases from a single UK paediatric transplant centre, who developed non-haematological alloimmune manifestations post HSCT.

### Patient 1

A 11 year old female with primary refractory acute lymphoblastic leukaemia (ALL) with JAK2 rearrangement in first remission underwent a fully matched unrelated donor (MUD) bone marrow transplant (BMT) with total body irradiation (TBI) and etoposide conditioning with alemtuzumab serotherapy. She engrafted with full donor chimerism by short tandem repeat (STR) and continued to be in molecular remission post HSCT. She developed grade 1 biopsy-proven gut graft-vs-host disease (GVHD) which was not treated. On day + 28 she developed human herpesvirus 6 (HHV-6) infection, detected in stomach biopsy (CT value 26), blood (positive, CT value not quantified) and CSF (positive, CT value not quantified), which was treated with ganciclovir. No other viruses were detected on CSF at that time.

Three months post-transplant the patient developed urinary retention which progressed to symmetrical ascending sensorimotor neuropathy up until T10 spinal root level. Seven weeks prior to her presentation she was SARS-CoV-2 positive which did not require treatment. On investigation, magnetic resonance imaging (MRI) spine indicated thickening and enhancement of the left-sided cauda equina nerve roots at L3/4 vertebral levels. Cerebrospinal fluid (CSF) analysis demonstrated elevated protein (0.55 g/L, peak 0.78 g/L 2 weeks later), normal glucose (2.6mmol/L), and elevated lactate (2.5mmol/L). Her CSF white cell count was 5 cells/uL, red cell count < 3 cells/uL, consistent with cytoalbumin dissociation as seen in Guillian-Barre syndrome (GBS). She had no positive CSF viral or antibody results. Her clinical presentation and investigations were in keeping with a diagnosis of GBS, however nerve conduction studies were not performed at the time of diagnosis. She did not have autonomic instability or require intensive care admission.

The patient received a course of intravenous immunoglobulin (IVIG) and pulse steroids, and had functional improvement, though, has persistence of intermittent urinary and faceal incontinence.

### Patient 2

A 16 year old male with high risk acute myeloid leukaemia (AML) with t(6;9) DEK::NUP214 rearrangement and FLT3-ITD mutation in first remission, underwent a 7/8 MUD umbilical cord blood (UCB) transplant with busulfan and fludarabine conditioning. He engrafted with complete donor chimerism and the disease continued to be in molecular remission. He was treated with steroids and extra corporeal photoperesis (ECP) for stage 3 acute skin GVHD.

**Fig. 1:**
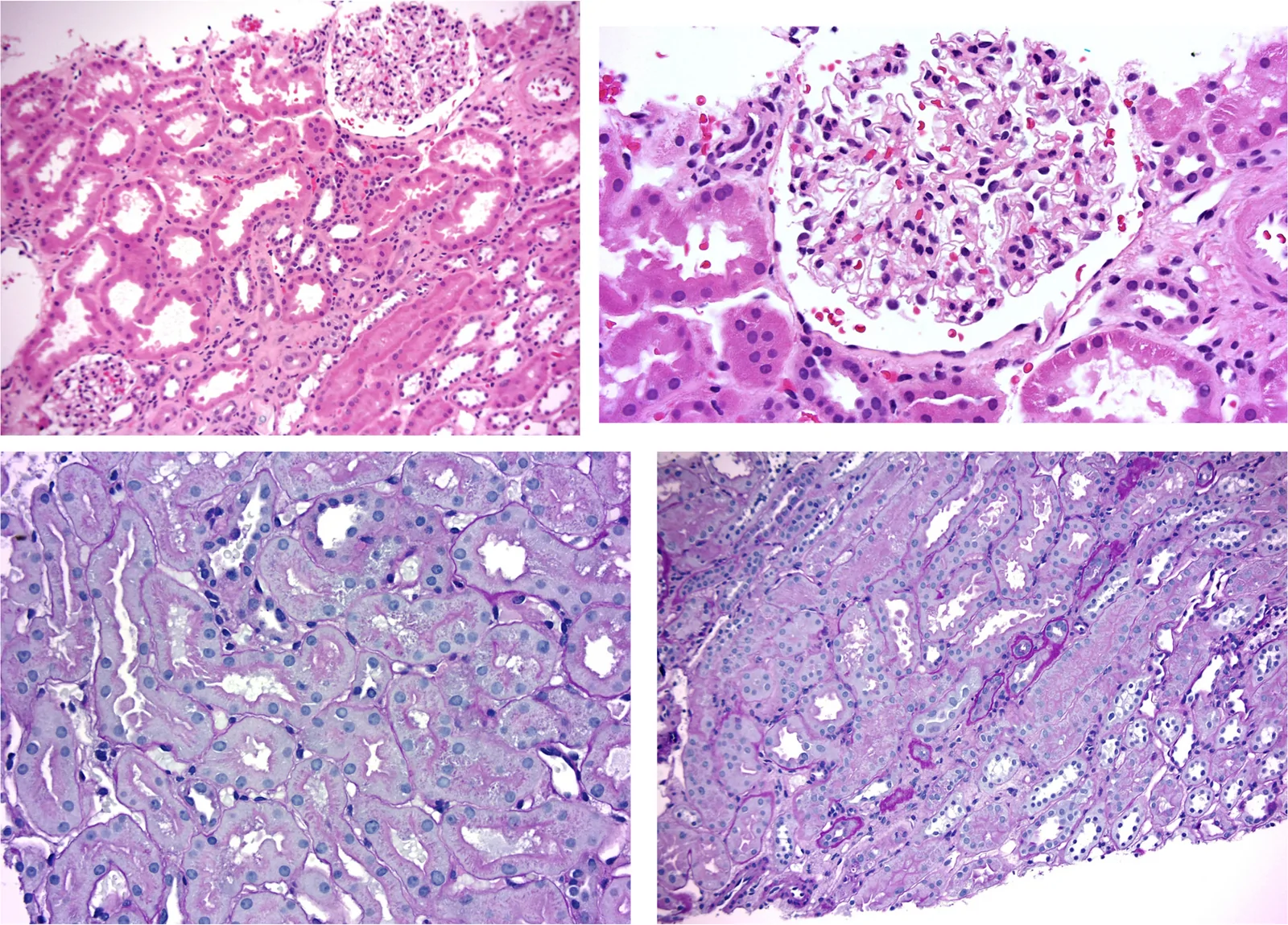
Renal biopsy from patient 2. Image 1 (top left) – H&E stain showing one glomeruli with glomerulomegaly and one shrunken glomerulus; Image 2 (top right) – H&E stain showing mild increase in mesangial cellularity; Image 3 (bottom left) – PAS stain showing dilated tubules with patchy loss of brush border; Image 4 (bottom right) – PAS stain showing thickening of tubular basement membranes

Two months post-transplant the patient developed acute onset hypoalbuminaemia (serum albumin of 11 g/L) with nephrotic range proteinuria (> 10,000 mg/L), and secondary peripheral edema. He was normotensive and the renal function remained unaltered. Kidney biopsy demonstrated mild to moderate tubular atrophy with patchy acute tubular damage, consistent with membranous nephropathy (see Fig. 1). Immunohistochemistry staining was absent for IgG, IgM, IgA, C3 and C1q. Other infectious causes for membranous glomerulopathy were excluded on analysis of biopsy sample, and his drug history was remarkable for routine ciclosporin use only as immunosuppression following transplant. The patient did not experience any other alloimmune manifestations.

The patient’s nephrotic syndrome rapidly responded to increased immunosuppression with rituximab, tacrolimus and steroids.

### Patient 3

A 2 year old male with relapsed AML with inv(16) in 2nd remission underwent a fully MUD donor bone marrow transplant with busulfan and cyclophosphamide conditioning with anti-thymocyte globulin (ATG) serotherapy. The patient engrafted with complete donor chimerism, and disease continued to be in molecular remission post HSCT. The patient developed stage 3 skin GVHD initially treated with steroids and ECP which rapidly resolved with the treatment given. At the same time, he experienced asymptomatic primary Epstein Barr virus (EBV) reactivation with the EBV PCR detected at 144,000 copies per ml in routine surveillance.

**Fig. 2:**
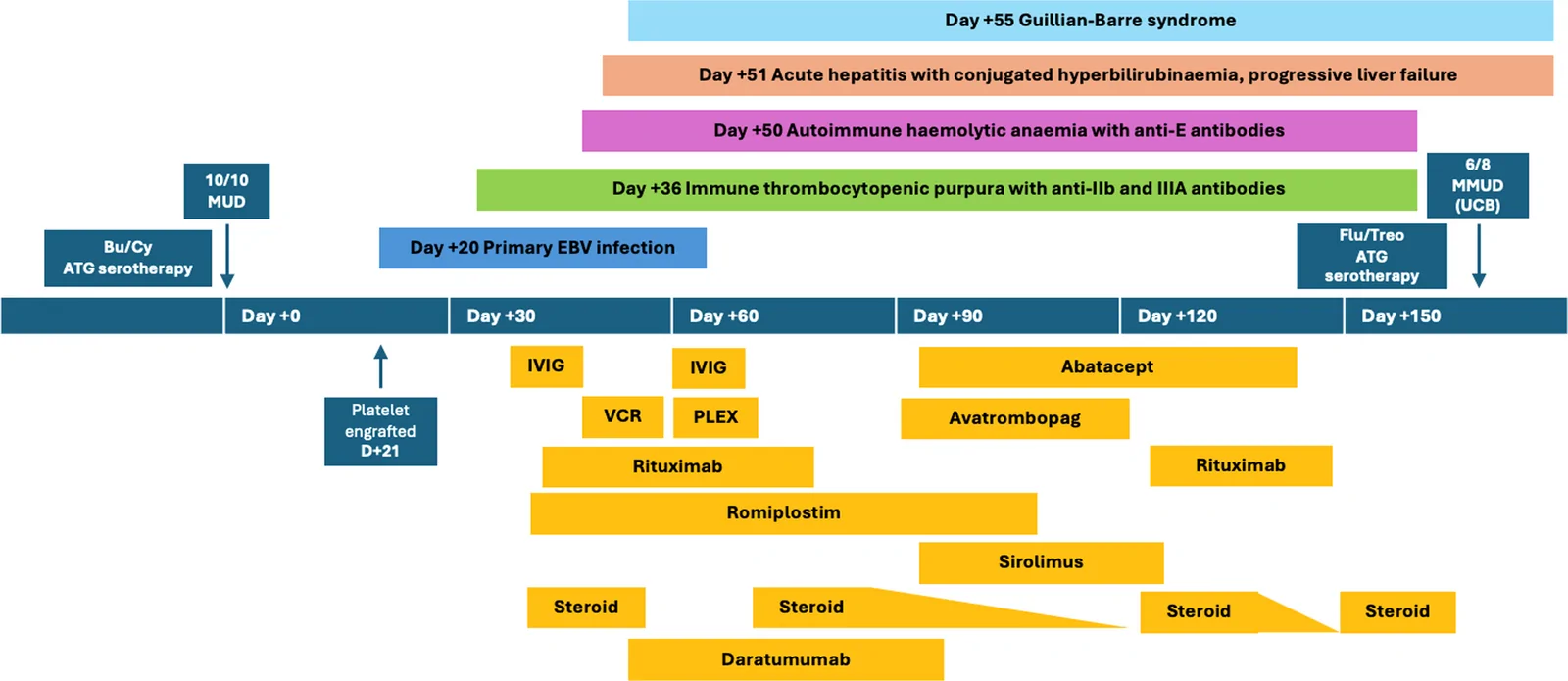
Patient 3 course and treatment. Bu-Cy – busulfan/cyclophosphamide; ATG – anti-thymocyte globulin; MUD – matched unrelated donor; EBV – Epstein-Barr virus; IVIG – intravenous immunoglobulin; PLEX – plasma exchange; VCR – vincristine; MMUD – mismatched unrelated donor; Flu/Treo – fludarabine/treosulfan; UCB – umbilical cord blood

As steroids were weaned by day + 40, the patient initially developed immune thrombocytopenic purpura (ITP), with detectable anti-GP IIb and IIIa autoantibodies. Shortly after on day + 50 he developed autoimmune haemolytic anaemia (AIHA) with anti-E, refractory to multiple lines of successive therapy including IVIG, steroids, rituximab, romiplostim, sirolimus, avatrombopag, abatacept, daratumumab, bortezomib and vincristine (see Fig. 2). There were no convincing features of transplant associated thrombotic microangiopathy (TA-TMA) including an elevated serum C5b-9 level. He was treated with a six week course of debriotide therapy for treatment of veno-occlusive disease however serial ultrasounds demonstrated normal hepatopetal flow in the portal vein and defibrotide treatment did not change his clinical picture. The patient was transfusion dependent and became progressively haemorrhagic with intermittent melaena despite HLA-matched platelet transfusions.

On day + 55, the patient developed acute onset muscle weakness, ptosis and bulbar palsy with respiratory failure requiring invasive mechanical ventilation in the intensive care unit. Imaging was unremarkable, and CSF analysis showed raised CSF protein, with anti-glutamic acid decarboxylase (GAD) antibodies. Nerve conduction studies were suggestive of acute sensorimotor axonal neuropathy (GBS, Miller-Fischer variant). Fludarabine-related neurotoxicity was considered as the patient had received a course of fludarabine and cytarabine for AML therapy 2 months prior to transplant, however the patient’s symptoms were not consistent with the typical clinical picture associated with fludarabine neurotoxicity and not consistent with the typical temporal course, with GBS symptoms developing 6 months following initial administration of fludarabine. The patient’s symptoms partially responded to pulse steroids, IVIG and plasma exchange, aiding him to be successfully extubated. However, he remained significantly deconditioned and non-ambulatory.

Subsequently, the patient had a significant transaminitis (peak alanine transaminase (ALT) 4506 IU/L) with conjugated hyperbilirubinaemia (serum bilirubin 356umol/L), and extensive work up for infection and metabolic causes was negative, with no evidence of autoantibodies. Liver biopsy was deemed risky given his significant, refractory ITP. Fibroscan demonstrated no evidence of cirrhosis or portal hypertension. Partial and brief improvement with liver enzymes was seen with abatacept and sirolimus.

Donor chimerism by STR remained 100% and the patient continued to be in molecular remission.

The patient had exhausted all available treatment options (see Fig. 2), and the decision was made to proceed to a second reduced intensity stem cell transplant due to significant and life threatning multi-system alloimmunity. This second transplant was delayed as long as possible following the first to reduce the significantly elevated risk of morbidity and transplant-related mortality associated with a second transplant. The patient was still recovering from significant morbidities and GBS. The patient unfortunately died on day + 5 following the second transplant due to acute multiorgan failure secondary to severe veno-occlusive disease.

## Discussion

Alloimmune manifestations following HSCT are increasingly recognised to be a complication impacting on overall survival and morbidity. Most commonly reported are autoimmune cytopenias, with an estimated incidence of up to 22% of paediatric HSCT complications [2].

Non-haematologic manifestations of alloimmune disease post-transplant, such as thyroid disease, neurological and peripheral nerve disease, autoimmune hepatitis, kidney disease and skin diseases, are rare and poorly described in paediatric patients in literature. They are important complications to identify and treat aggressively, as they are often treatment-refractory and associated with a poor prognosis. Risk factors known to be associated with these alloimmune conditions are non-malignant indication for HSCT, use of an unrelated donor, omission of TBI in conditioning, presence of cGVHD, or peripheral blood stem cell (PBSC) or UCB donor source [3]. The incidence of non-haematologic manifestations of alloimmunity in paediatric patients is exceedingly rare, with less than 10 cases reported of GBS, and 1 case of autoimmune hepatitis [1].

The underlying pathophysiology of alloimmune disease following transplant is not well understood. Innate immune reconstitution occurs within the first few weeks following transplant, with restoration of neutrophils, natural killer cells, monocytes and dendritic cells first [4]. This is followed by reconstitution of the adaptive immune system, where donor CD8 memory T cells expand to respond to stimulation from cytokines or viruses, and later recovery of thymic function to enable differentiation of CD4 and CD8 T cells to be self-tolerant, which may take up to a year following transplant. Critically, regulatory T cells (Tregs) are essential in suppressing effector T cells to reduce inflammation and promote immune tolerance [5] and also keeps the B cells in check from a dysregulated polyclonal expansion and the resultant autoantibody production.

The pace of immune reconstitution is sensitive to factors associated with the transplant process, including conditioning regime, donor source, graft manipulation, presence of graft-vs-host disease and infection. Use of in-vivo T cell depletion with ATG or alemtuzumab serotherapy, or ex-vivo T cell depletion strategies of the graft, may prolong T cell recovery and dysregulate the fine balance between B and T cell immunity (see Table 1 for patient-specific immune reconstitution patterns). Viral infections have been implicated in the development of autoimmunity post-transplant via molecular mimicry, and case reports of patients developing GBS post HHV6 infection have been described, such as in the case of Patient 1 [6]. There is also emerging evidence of causative association between SARS-CoV-2 infection and alloimmunity by virtue of triggering such a process by molecular mimicry [7]. Patient 1 experienced GBS after a few weeks of asymptomatic SARS-CoV-2 infection, and treated HHV-6 infection, which might be relevant in causation.

**Table 1:** Immune reconstitution patterns in the three patients. Reference ranges - CD3 800–3500 cells/uL, CD4 400–2100 cells/uL, CD8 200–1200 cells/uL, CD19 200–600 cells/uL, CD16/CD56 70–1200 cells/uL. MUD – matched unrelated donor, UCB – umbilical cord blood, ATG – anti-thymocyte globulin

		Patient 1 (MUD source, alemtuzumab serotherapy)	Patient 2 (UCB source, no serotherapy)	Patient 3 (MUD source, ATG serotherapy)
At diagnosis of alloimmune manifestation	CD3	192	294	190
	CD4	172	242	44
	CD8	20	40	131
	CD19	54	654	9
	Absolute CD16/CD56	131	168	123
At 6 months	CD3	200	251	113
	CD4	105	212	26
	CD8	108	34	85
	CD19	205	226	< 1
	Absolute CD16/CD56	161	124	19
At 12 months	CD3	674	533	N/A
	CD4	180	399	N/A
	CD8	173	114	N/A
	CD19	246	401	N/A
	Absolute CD16/CD56	116	143	N/A

**Fig. 3:**
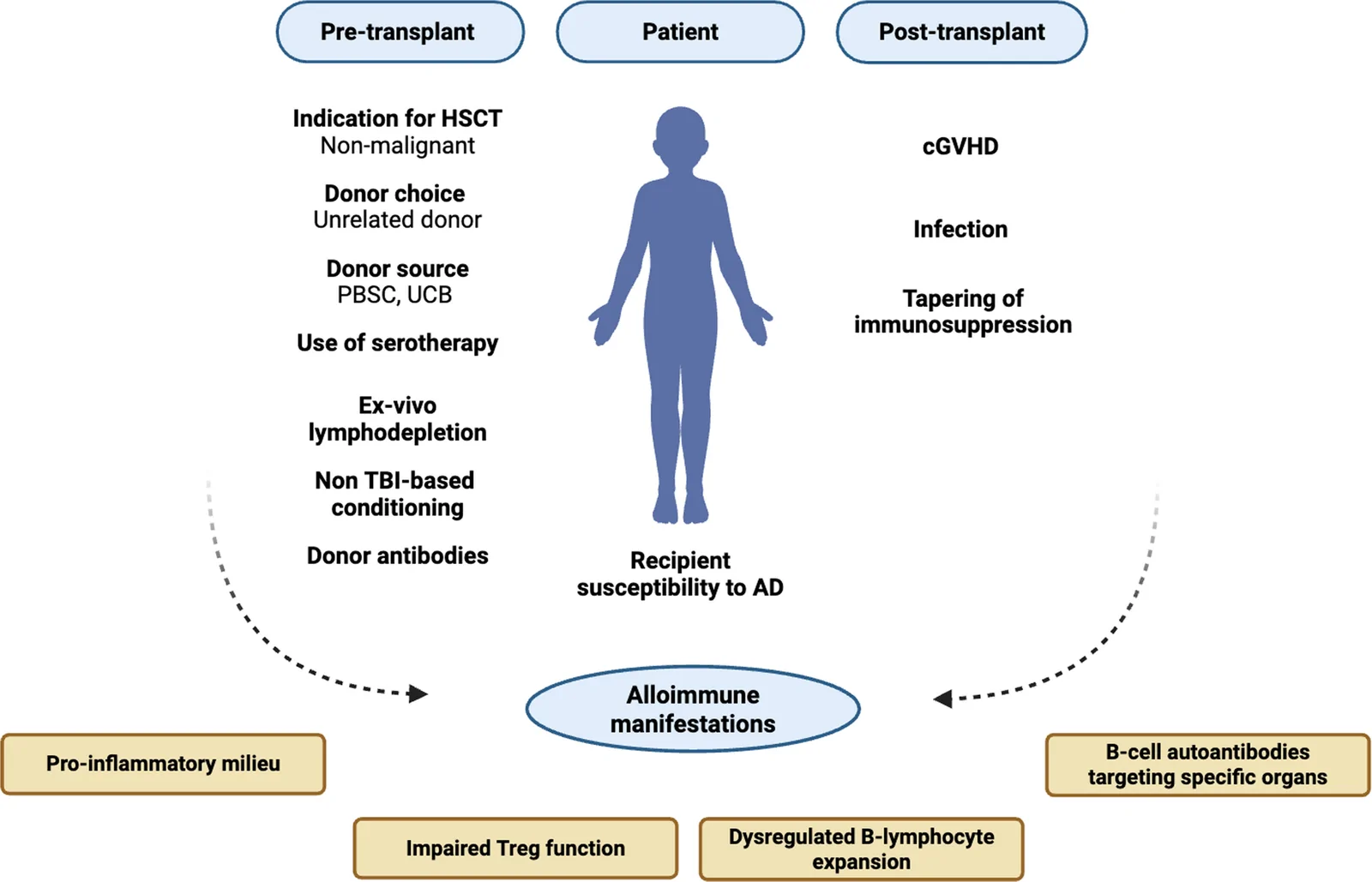
Risk factors associated with the development of alloimmune manifestations post transplant and contributing pathophysiology. PBSC – peripheral blood stem cell; UCB – umbilical cord blood; TBI – total body irradiation; AD – alloimmune disease; cGVHD – chronic graft-vs-host disease; Treg – T-regulatory cells

Impaired or dysfunctional immune tolerance during immune reconstitution, mediated by Treg cells, can lead to unregulated B lymphocyte expansion and a pro-inflammatory cascade [1]. Additional post-transplant pro-inflammatory factors such as viral infection can contribute to this dysfunction, leading to B cell alloantibody production (see Fig. 3). Donor immune response to donor antigens, targeted at donor red blood cells, platelets, or neutrophils, drives autoimmune cytopenia. Shared antigens between the donor and host are the target of donor autoantibodies on specific organs, leading to non-haematological manifestations of autoimmunity, including autoimmune thyroid disease and myasthenia gravis (MG) [1]. Alloimmune-mediated renal disorders such as membranous nephropathy are due to subepithelial deposition of donor-derived immune complexes in the glomerular basement membrane, such as in the case of Patient 2, and typically develop following the discontinuation of immunosuppressive therapy [8, 9]. Host-related genetic predisposition to autoimmune disease, and donor transfer of autoantibodies, may also contribute to development of alloimmune manifestations [10, 11].

Whether post-transplant alloimmunity represents an extended spectrum of cGVHD, sharing the same pathophysiology, remains an area of research. While GVHD is also an immune-mediated process, it is predominantly a T-cell mediated process and it typically targets tissues associated with the immune barrier, including the skin, gastrointestinal tract and respiratory system, rich in antigen presenting cells (APC) such as dendritic cells and macrophages. It resembles an autoimmune disease, however, does not exhibit development of autoantibodies against specific tissues. It is possible that some of the manifestations of alloimmune disease post-transplant may reflect a spectrum of chronic GVHD [9, 12].

This case series demonstrates the diversity of non-haematologic alloimmune manifestations in paediatric HSCT patients. 2/3 patients received serotherapy with ATG and alemtuzumab, likely contributing to prolonged T cell suppression. Patient 1 experienced HHV6 viraemia and SARS-CoV-2 infection prior to development of GBS, while Patient 3 had primary EBV reactivation, possibly triggering further dysfunction in a growing immature immune system. All three patients had preceding aGVHD which was responsive to treatment and well controlled, and none experienced cGVHD. Reflecting the challenges of treating such conditions, all 3 patients required multiple lines of therapy to induce remission of their autoimmune disease. Patient 3, in particular, experienced an unusual combination of multi-system disease, as yet unreported in the literature, requiring a second HSCT to manage complications associated with significant alloimmune disease. No donors were identified to have significant autoimmune disease. All patients experienced significant morbidity associated with their alloimmune diseases, highlighting the need for early identification and prompt treatment of symptoms.

## Conclusion

Whilst rare, non-haematologic alloimmune manifestations following transplant are clinically significant and often refractory to treatment. Diagnosis can be challenging due to similar clinical features seen in other more common HSCT complications. Further understanding of the pathophysiology of such conditions is essential to identify patient-specific risk factors and allow for early diagnosis and initiation of targeted therapies to improve patient outcomes. Our reported experience adds to the range of systemic alloimmunity documented in literature, giving insightful consideration for early recognition and treatment.

## Data Availability

No datasets were generated or analysed during the current study.
